# The Role of Interfacial Adhesion on the Mechanical Behavior of Thin Metal/Polymer Laminate with Surface Roughness

**DOI:** 10.3390/polym14153131

**Published:** 2022-07-31

**Authors:** Mahdieh Shahmardani, Rafat Mohammadi

**Affiliations:** 1 Department of Mechanical Engineering, Faculty of Engineering, Arak University, Arak 38156-88349, Iran; 2 Interdisciplinary Centre for Advanced Materials Simulation (ICAMS), Ruhr-Universität Bochum, Universitätsstraße 150, 44801 Bochum, Germany

**Keywords:** interfacial adhesion, thin Al/LDPE laminate, surface roughness, damage properties

## Abstract

Metal/polymer laminate has versatile applications in industry due to the essential roles of its constituents in controlling its mechanical behavior. Therefore, efforts to enhance a laminate’s performance should target its mechanical behavior. One of the most influencing features of the mechanical behavior of this type of thin laminate is the interface layer properties. This study concentrates on the mechanical response of thin aluminum (Al) foil deposited on a polymer substrate by calibrating its interfacial layer properties based on available uniaxial tensile tests performed on thin Al/polymer laminate. Then, taking into account the calibrated parameters for the interface layer, which leads to mimicking the real conditions of the laminate, one type of imperfection is introduced as a wavy roughness on the surface of each layer with different amplitudes to investigate its influence on the overall mechanical behavior of the laminate and its failure mode. The results highlighted that the existence of the roughness on the surface of the polymer layer reduces the maximum engineering stress of the laminate more severely compared to other conditions. As the roughness amplitude increases, the maximum stress reduces a lot. The distribution of equivalent plastic strains represents the appearance of the shear bands in the Al layer and an almost uniform distribution for the polymer layer. In the case of existing roughness on each layer, a higher amount of plastic strain accumulation occurs in the middle of the polymer layer and top corners of the thin Al layer. Due to the significant effect of interfacial layer properties to improve the maximum strength of the laminate and its final elongation, a parametric study is performed, taking into account different interfacial properties. The results indicate that laminate behavior with weaker separation properties in the interface layer is mostly unaffected by adopting higher tractions, and no change happens in the case of high separation considering weak tractions.

## 1. Introduction

Metal composites have attracted much attention due to their vast applications. One of the applications of metal composites is flexible electronics, which are produced by assembling electronic circuits and mounting electronic gadgets on flexible plastic substrates made of polyimide or transparent conductive polyester [1]. Applications concern flexible (rollable) displays, printable thin film solar cells, paper-like displays and electronic (artificial) skins [2]. To make the structure stretchable, all the components must comply with some degree of bending or a specific percentage of stretch without losing their capability to function. The functional appropriateness of flexible electronics strongly depends on the adhesion of the metal/polymer interface as well as on the geometry of the metal and polymer layers separately. Using numerical modeling, two competing failure mechanisms (metal film necking versus grain boundary cracking) of thin metal films on polymer substrates were studied [3]. They showed that when the grain boundaries of the metal films are weak, the thin metal films tend to rupture by grain boundary cracking. On the other hand, when the grain boundaries of the metal films are strong, then the thin metal films will mainly be ruptured by necking. The results indicated that the metal film ductility is modulated by the metal/polymer interfacial adhesion but generally ranges from low to modest. The quantitative results suggested that to achieve high ductility of thin metal films on polymer substrates, it is desirable to have strong grain boundaries of the metal films and strong metal/polymer interfacial adhesion. A finite element method was used [4] to simulate debonding of the metal/polymer interface and necking of the metal film. The metal/polymer interface was modeled as nonlinear springs specified by a tensile and shear traction–separation (T–S) law. Depending on interface properties, three types of tensile behavior were identified, and they showed that the rupture strain and necking of the metal film modulate by interfacial sliding rather than interfacial opening.

Some experimental tests were conducted to investigate the basic mechanism of metal film attached to a polymer substrate, and annealed Cu coatings supported by polyimide substrates were simulated by finite element methods [5]. They found out the failure strain of a metal film on a polymer substrate could be maximized by ensuring good adhesion between the film and the substrate, adopting a uniform crystallographic texture, large grain size and low yield strength. In another study, the strain delocalization in a weakly hardening metal film on a steeply hardening polymer substrate was studied [6]. They used a finite element code to simulate large-amplitude non-uniform deformation in both freestanding and substrate-bonded metal films. Two debonded lengths were specified to investigate their effect on the rupture strain of the metal, and the results showed that the longer the debonded length in use, the smaller the rupture strain. 

Two sets of Cu films deposited on polyimide substrates with good and poor adhesion were tested experimentally [7] to demonstrate the role of film/substrate adhesion on the ductility of thin metals. A well-bonded substrate carried the Cu layer to large tensile strains without rupture by delocalizing the deformation in the film. A poorly bonded substrate allowed the metal to form channel cracks at relatively small macroscopic strains by facilitating the coevolution of strain localization and delamination. 

Due to the thinness of the metal film, the influence of wrinkling becomes crucial in these laminates [8]. The numerical modeling of the laminate was performed using a micro-material model based on the experimental state of the material and the effect of different characteristics, such as a wrinkle on creased laminate, ply numbers and thickness of layers, under in-plane shear deformation with different load variations, were studied. The results highlighted the significant impact of the layer thickness on laminate failure.

Another application of metallic composites is food packaging systems. Food packages have become part of our daily life. Due to the development in the packaging industry, it is now possible to buy well-stored food produced thousands of miles away. At the same time, packaging companies, as well as consumers, have started to pay attention to societal sustainability aspects. Using less raw materials, reducing costs and developing package functionalities are today’s goals of research and development. Any sudden change in the role of laminate layers may cause catastrophic effects on people’s health. In this regard, versatile studies have been performed on this type of thin laminate to improve its effectiveness in the mentioned application [9,10,11]. A new experimental technique was also developed to distinguish the defects in the fabricated laminate that are not detectable by the observer’s view and may lead to catastrophe failure [12].

The mechanical properties of the Al foil, low-density polyethylene (LDPE) and the influences of their adhesion were investigated by analytical and finite element methods [13]. By performing tensile tests and using analytical formulas, the authors calculated Young’s modulus of the Al and LDPE. Finite element computations were also carried out by ABAQUS with an elastic-plastic Mises model considering isotropic hardening for each layer. The results showed that peak stress and the corresponding strain increase with the adhesion level, and a higher adhesion level leads to higher peak stress in the laminate. In [14], the failure behavior of thin freestanding Al foil under a uniaxial tensile test was evaluated, and the authors could reproduce a failure mechanism of Al foil similar to the experimental test behavior using a numerical method. The results of this study are crucial for a better understanding of the mechanical behavior of Al foil in Al/polymer laminate.

In laminated structures, the influences of cracks are significant, and it is essential to know the fracture characteristics of a crack along or perpendicular to the laminate interface. To study these characteristics, analytical methods were used to derive basic equations for cracks perpendicular to the interface of a finite and infinite solid [15]. The dislocation simulation approach as a series of the first Chebyshev polynomial showed that for the crack situated in weaker material, the stress intensity factor is smaller than that in the homogeneous material and larger for cracks in a stiffer material. They observed that for the finite and infinite solids of biomaterial, despite the existence of different stress intensity factors, the stress distributions were similar. Another research studied the micro-mechanisms of fracture in a laminate composed of an Al foil and a polymer film [16]. A micro-mechanical approach utilizing scanning electron microscope (SEM)-micrographs, micro-mechanisms and an analytical expression motivated the derivation of an equation suitable to calculate the work of failure of freestanding and laminated thin Al foil. The authors applied a tensile test on the thin laminate of Al foil and LDPE to evaluate their stress–strain relationships. With the assumption of perfect adhesion between these two layers, LDPE could increase the rupture strain of Al foil to a reasonable extent.

The mechanical and fracture behavior of a thin Al foil and polymer laminate was studied by [17]. The possibility of controlling the path of the growing crack propagation by adjusting the adhesion level and the property of the polymer layer was investigated. First, the fracture process of the Al foil was studied experimentally. Secondly, the mechanical and fracture behavior of the laminate were evaluated. A theory for the mechanics of the composite material was used to evaluate a series of experiments. Then, the behavior of a crack in one of the layers, perpendicular to the bi-material interface in a finite solid was evaluated by formulating a dislocation superposition method. The results showed that the analytical methods for an asymptotically small crack extension could also be applied for a fairly large amount of crack growth. As adopting a proper set of material parameters for each layer in the laminate leads to realistic results, the fracture properties together with damage parameters of the laminate and the adhesion between two layers were defined based on experimental and theoretical methods by [18]. 

The opening procedure in the packaging system was also studied in [19], which simulated a numerical model including realistic material behavior from experimental tests and considering large deformations and fracture mechanic concepts. The result of the center-cracked tension specimen under fracture mode I was compared with the experimental test and showed good agreement. Various models were developed [20] to recover the adhesion properties of the Al/polymer laminate and highlighted the difficulties in characterizing the interface properties. 

In the above studies, different investigations indicated the important role of interfacial adhesion properties on the mechanical behavior of the laminate, which makes it necessary to obtain those properties comparable with experimental data to be applicable in real conditions. In the current study, first, the interface properties are calibrated considering the bi-linear T–S law and based on the available uniaxial tensile test on the laminate. Then, the influence of surface roughness as a kind of imperfection on the mechanical behavior and failure of laminates is studied. The surface roughness is assumed to be sinusoidal, and the effects of its wavelength and amplitude are investigated independently. The details of the interfacial layer properties are investigated in a complete parametric analysis due to their considerable significance. The development of the numerical model of the adhesion layer with its properties like the real laminate condition is an advancement to study this kind of thin laminates’ mechanical behavior numerically. With the knowledge of interface layer properties and the adoption of possible imperfection in numerical models, the results of the current study are useful in real applications.

## 2. Materials and Numerical Methods

The materials used in the current study consist of thin Al foil deposited on an LDPE layer, and their mechanical behavior together with material properties are described in the following.

### 2.1. Kinematics and Constitutive Law

Based on the continuum mechanic theory for elastoplastic materials behavior [21], logarithmic strain defined in the numerical model is formulated based on large deformation theory as:

(1) $$\varepsilon =\sum_{i=1}^{3}\ln\left(\lambda_{i}\right)N_{i}⊗N_{i},$$

where $\lambda_{i}$ is the principle stretch in principle direction of $N_{i}$. The logarithmic strain can be related to the rate of deformation by:

(2) $$\dot{\varepsilon }=sym\left(\dot{F}\cdot F^{-1}\right),$$

where **F** is the gradient of deformation. With the assumption of small elastic strain and large plastic strain, the total strain rate can be decomposed additively as

(3) $$\dot{\varepsilon }={\dot{\varepsilon }}^{el}+{\dot{\varepsilon }}^{p},$$

where ${\dot{\varepsilon }}^{el}$ and ${\dot{\varepsilon }}^{p}$ are elastic strain rate and plastic strain rate, respectively, in which the response with small elastic strain is formulated as

(4) $$\sigma =C\;\varepsilon^{el},$$

where C is the elastic stiffness matrix. The elastic domain is restricted by a yield surface in the form of

(5) $$f\left(\sigma ,\bar{\sigma }\right)=0,$$

where $\bar{\sigma }$ corresponds to the evolved yield limit considering exponential hardening according to relationship

(6) $$\bar{\sigma }=\sigma_{0}{\left(1+\frac{E{\bar{\varepsilon }}^{p}}{\sigma_{0}}\right)}^{n},$$

where $\sigma_{0}$ is the initial yield surface, and *n* is the hardening exponent. ${\bar{\varepsilon }}^{p}$ represents the equivalent plastic strain during plastic deformation

(7) $${\bar{\varepsilon }}^{p}=\sqrt{\frac{2}{3}{\left(\varepsilon_{xx}^{p}\right)}^{2}+\frac{2}{3}{\left(\varepsilon_{yy}^{p}\right)}^{2}+\frac{1}{3}{\left(\varepsilon_{xy}^{p}\right)}^{2}},$$

and with the associated material response, plastic strains in relationship 7 are defined by the following flow rule:

(8) $${\dot{\varepsilon }}^{p}=\dot{\Lambda }\frac{\partial f}{\partial \sigma },$$

where $\dot{\Lambda }$ is the plastic multiplier defining the loading/unloading criterion through the complementary conditions:

(9) $$f\leq 0,\;\;\;\dot{\Lambda }\geq 0,\;\;\;\dot{\Lambda }f=0.$$

### 2.2. Finite Element Model

To investigate the mechanical behavior of the laminate numerically, finite element models are developed that implements both material and geometrical nonlinearity. An elastic-plastic constitutive law based on the classical Huber–Hencky–Mises yield criterion is assumed for Al and LDPE. True stress versus the logarithmic strain curve of Al and LDPE, extracted from the experimental tensile tests performed by [16] are used as the material’s behavior for numerical simulations. The mechanical properties of Al and LDPE are listed in Table 1.

The problem domain is discretized by four-node and eight-node elements with randomly distributed, fine meshes considering either two-dimensional (2D) or three-dimensional (3D) quasi-static analyses, respectively. The plane stress element is used for the 2D problem, and solid elements are adopted for 3D analyses. The clamped end boundary conditions are applied on one end, and the loading is applied on the other end in the strain-controlled condition.

### 2.3. Material Parameters Calibration 

**Figure 1:**
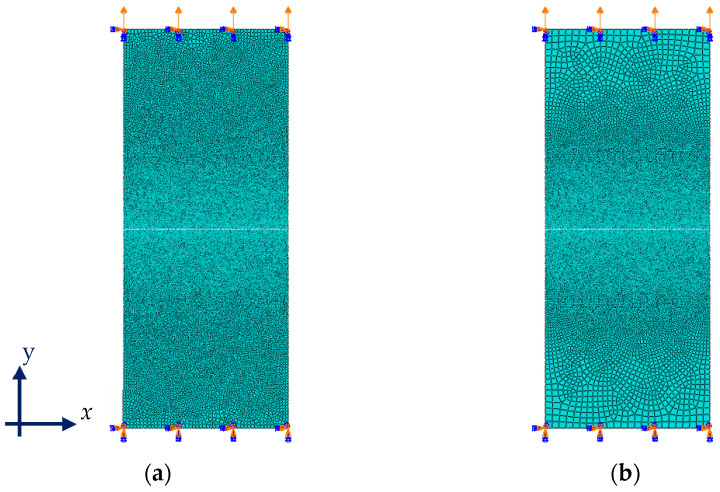
Boundary conditions and random mesh discretization of (**a**) plain and (**b**) center cracked laminates.

To model the softening behavior of the laminate, an interface layer is considered between laminate layers. Since the focus here is to calibrate the interface properties, the laminate is modeled numerically considering its average mechanical behavior [16] as a homogeneous solid structure and by using plain stress conditions and neglecting its composition of Al foil and LDPE layers separately. The average mechanical properties of the laminate are listed in Table 2. The geometry of the plain and notched laminates is the same as the tested samples in experiments performed by [16] and is listed in Table 3. The discretized 2D numerical model of the laminate visualizing its boundary condition is depicted in Figure 1. The details of experimental tests on the thin laminate are discussed completely in [16].

**Figure 2:**
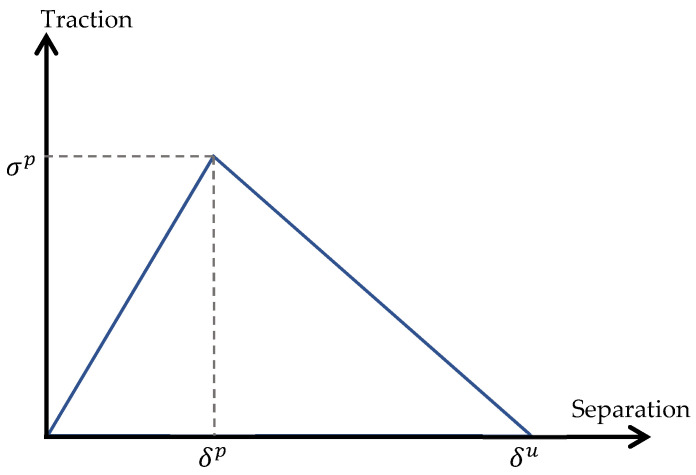
The T–S law is used to model the interface behavior.

The interface layer is modeled by zero-thickness cohesive elements, and its behavior is characterized by a bilinear T–S law (visualized in Figure 2), whose parameters are determined by calibrating based on the behavior of the plain laminate samples under uniaxial load in the experimental test.

The failure mechanism represented in Figure 2 is characterized by three parameters; the peak traction stress $\sigma^{p}$, the corresponding separation displacement $\delta^{p}$ and the ultimate separation displacement or displacement jump when decohesion occurs $\delta^{u}$. The same normal and shear tractions are considered in the following study, and separations are assumed to be normal to the interface and in the shear direction, respectively. The initial stiffness of the cohesive zone model should be high enough to keep a stiff connection between two neighboring bulk elements and to obtain realistic results [22]. The initial stiffness is considered to be 1000 times that of Al foil Young’s modulus [23]. The maximum normal/shear strength is chosen from the maximum stress experienced in the nominal stress-strain curve of the laminate under tensile loading in experiments. The ultimate separation parameter, which describes the strain localization and the softening branch of the laminate, is determined iteratively to capture the strain localization of the plain sample acquired by a tensile machine. The calibrated interface properties are listed in Table 4.

**Figure 3:**
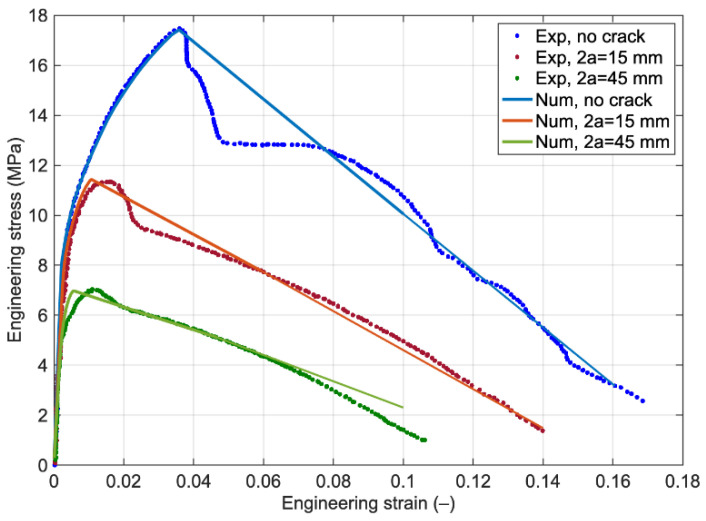
Comparison of the engineering stress-strain curve of plain and notched laminates obtained by experimental test and numerical methods using interface layer.

Figure 3 shows the engineering stress-strain behavior of the plain and notched laminates (with central crack lengths of 15 and 45 mm) under tensile loading by calibrating the interfacial properties based on the plain samples from the experimental test. The predicted curves of two notched laminates highlight that the numerical model could capture both the maximum nominal stress and the softening behavior. As visualized in Figure 3, in the experimental tests data, after maximum stress, the laminate first displays a softening behavior due to the degradation of the Al foil layer and then a plateau (in the case of the plain sample) and another softening branch with a different slope, which is supported seen by only a polymer layer. While in the numerical models of plain and notched laminates, after maximum stress, just one softening branch exists that is due to the modeling of the laminate based on its average mechanical behavior from experimental tests and neglecting the Al foil and LDPE layers behavior separately. 

## 3. Comparative Results

### 3.1. The Influence of Surface Roughness 

In the current study, as a kind of imperfection in the laminate, surface roughness is considered on the surface of the laminate layer and its impact is studied numerically. Here, we focus on the effect of the surface roughness of Al and the LFPE on the laminate’s overall behavior, taking into account the influence of interfacial damage using calibrated interfacial layer properties. 

**Figure 4:**
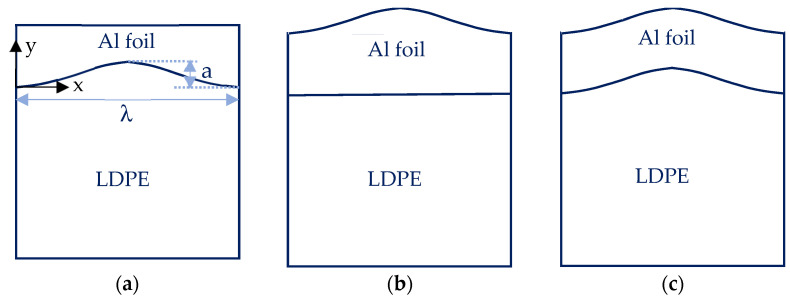
Polymer-supported Al film with an idealized sinusoidal interface: (**a**) wavy LDPE surface with flat Al surface; (**b**) wavy both Al and LDPE surface and (**c**) wavy Al surface with a flat LDPE surface.

For simplicity, it is assumed that the wavy surface has the following idealized sinusoidal form [24]:

*y* = *a* sin(*π*/*λ*)*x*, (10)

where *a* and *λ* denote the amplitude and wavelength, with the *x*-axis pointing to the right horizontally (see Figure 4a). Depending on how the thin Al film is deposited, three possible cases are envisioned to exist: (a) the Al surface is flat, as sketched in Figure 4a; (b) the Al surface is wavy, identical to that of the LDPE surface (Figure 4b) and (c) the Al surface is wavy, but the LDPE surface is flat (Figure 4c).

To investigate the effect of surface roughness on the overall behavior of the laminate, three different values for amplitude ratio (*a/λ*) are considered with three wavelengths (*λ*) equal to *h*/2, *h* and 2*h*, in which *h* is the Al foil thickness. Therefore, the ratio of *a*/*λ* in the numerical models is changed to 0, 0.025 and 0.05. The behavior of the laminate reveals that upon applying the load, necking occurs in the Al foil layer and the thickness of Al foil plays an important role in the appearance of this necking and final failure mode of the laminate. In this regard, the wavelength of the roughness was chosen to be proportional to the Al foil thickness.

**Figure 5:**
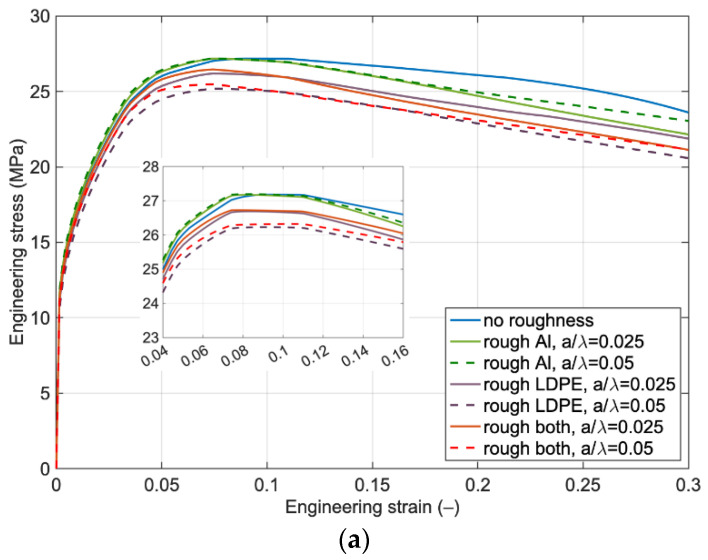

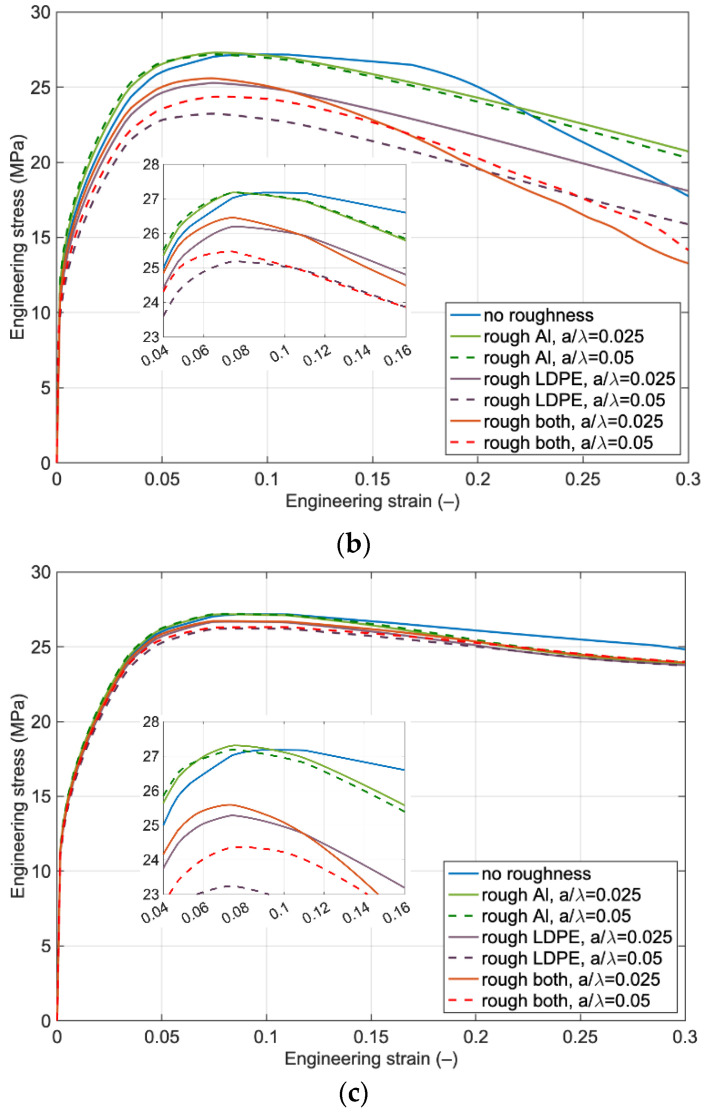
The effect of roughness on engineering stress versus engineering strain for the laminate with wavelength (λ) equal to (**a**) h/2, (**b**) h and (**c**) 2 h and different amplitudes.

Figure 5a represents the influence of surface roughness on Al and LDPE separately and on both surfaces with different amplitudes and *λ* equal to *h*/2. The results highlight that maximum stress occurs for the case of roughness only on the Al foil surface, and roughness only on the LDPE results in the lowest level of stress. Roughness also affects the softening branch such that compared to the without-surface roughness, the laminate degradation happens earlier.

Figure 5b depicts the effect of surface roughness with a wavelength equal to the Al foil thickness. The same behavior in the *λ* equal to *h*/2 is also observed here, but the rate of decrease in the stress level by changing the roughness from the Al foil surface to the LDPE surface is higher, and it will be magnified by higher amplitudes.

Increasing the wavelength leads to a significant difference in the overall behavior of the laminate with roughness on its layers. The results in Figure 5c indicate that a higher value of wavelength weakens the overall mechanical behavior of the laminate, and strain localization triggers at a much lower strain level. It is worth noting that in spite of the lower level of stress for the case of roughness on the LDPE layer, the anticipation of softening branch happens for the roughness on both Al foil and LDPE layers. 

**Figure 6:**
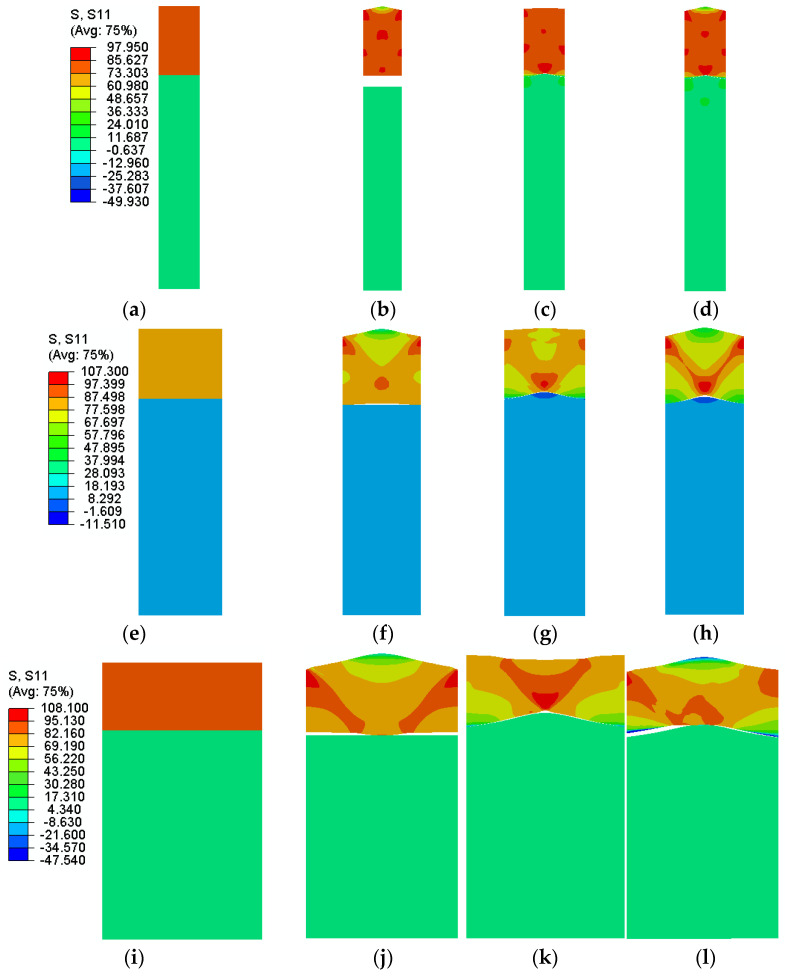
Distribution of stress parallel to the loading direction (𝑠11) at maximum stress for the laminate with a wavelength of (**a**–**d**) h/2, (**e**–**h**) h, (**i**–**l**) 2 h and (**a**,**e**,**i**) without roughness, (**b**,**f**,**j**) with wavy Al surface, (**c**,**g**,**k**) with wavy LDPE surface and (**d**,**h**,**l**) with both Al foil and LDPE wavy surfaces.

The distribution of stresses parallel to the loading direction (along the *x*-axis) at maximum nominal stress for different wavelengths is shown in Figure 6. In Figure 6a, there is no roughness on the surface of laminate layers, the stress in the Al foil layer is very high and its distribution is uniform. On the other hand, the stress value is very low at the polymer layer but still with a uniform distribution. The distribution of stresses in the laminate with roughness on Al foil, LDPE and both with the amplitude equal to 0.05 is visualized in Figure 6b–d. Surface roughness causes a change in the distribution of stress in the Al foil layer only, thus reducing stress, particularly at the surface but increasing stress at the edges. In addition, at the Al foil layer, stress concentration occurs at specific locations along the Al foil thickness. Applying higher elongation to the laminate can reveal the stress concentration more clearly by forming cross bands.

**Figure 7:**
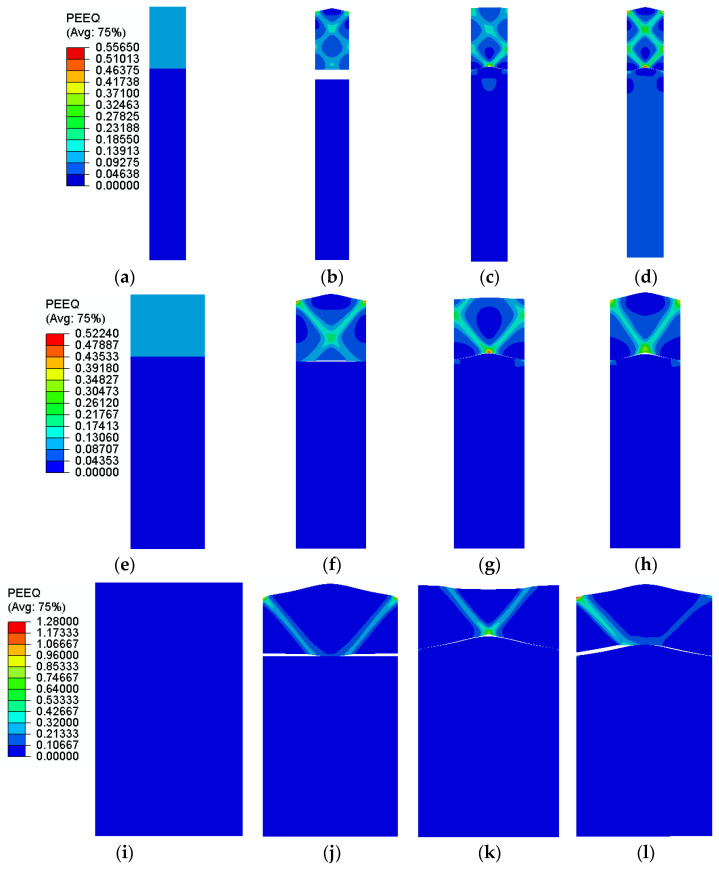
Distribution of equivalent plastic strain at maximum stress for the laminate with a wavelength of (**a**–**d**) h/2, (**e**–**h**) h, (**i**–**l**) 2 h and (**a**,**e**,**i**) without roughness, (**b**,**f**,**j**) with wavy Al surface, (**c**,**g**,**k**) with wavy LDPE surface and (**d**,**h**,**l**) with both Al foil and LDPE wavy surfaces.

The distribution of equivalent plastic strain at maximum stress is visualized in Figure 7 for a perfect laminate and laminate with different wavelengths. When there is roughness on the LDPE surface, the distribution of equivalent plastic strain will not remain constant at the polymer layer compared to the flat Al surface. Hence, in the presence of surface irregularities, the polymer layer accompanies Al foil to redistribute the applied strain. The results also highlight that in the presence of surface roughness, the strain will localize to create shear bands, as represented in Figure 7; by reducing the wavelength, the number of shear bands increases. In general, increasing the wavelength results in a higher amount of plastic strain in the laminate and comparing three different surface roughness conditions demonstrates that the laminate with roughness on both layers experiences severe plastic strain localization.

**Figure 8:**
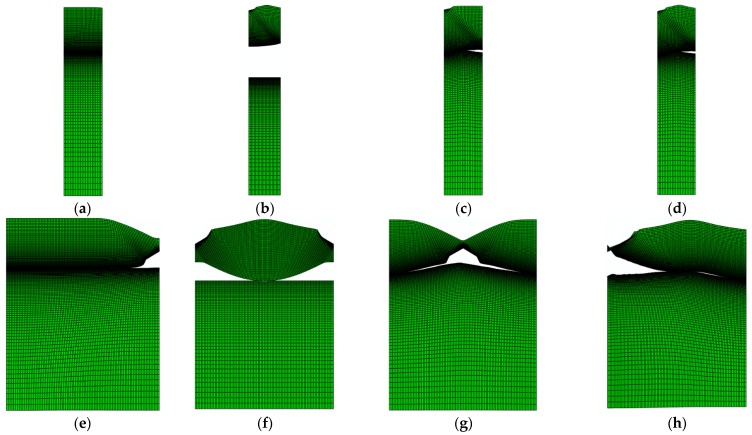
Failure mode of the laminate for two different wavelengths of (**a**–**d**) h/2 and (**e**–**h**) 2 h, (**a**,**e**) without roughness, (**b**,**f**) roughness on Al foil surface, (**c**,**g**) roughness on LDPE surface and (**d**,**h**) roughness on both layers.

The results in Figure 8 depict the influence of surface roughness on the failure mode of the laminate at the end of loading with two different wavelengths. As visualized in Figure 8a–d, the failure mode of the laminate with a wavelength of h/2 is almost the same for the case of roughness only on the Al foil surface (Figure 8b), on the LDPE surface (Figure 8c) and on both Al and LDPE surfaces (Figure 8d) in which there will be one shear band due to necking in the Al foil layer. On the other hand, in the case of no surface roughness, the laminate failure mode changes by increasing the wavelength, resulting in the emergence of necking in the Al foil layer (see Figure 8a,e). As visualized in Figure 8f–h, two neckings will be on both sides of the Al foil due to roughness on the Al surface, one necking in the middle of the Al foil layer with roughness on the LDPE surface (Figure 8g), and one side necking with multi-debonding along the length of the laminate interface when there is roughness on both Al foil and LDPE surfaces (Figure 8h).

### 3.2. The Influence of Different Interfacial Properties

This section investigates the influence of interfacial properties without imperfections (surface roughness). To study the effect of interface properties on the laminate behavior, different values for traction and separation are defined as listed in Table 5. Logically, the initial stiffness will change accordingly by changing the traction and separation values.

**Figure 9:**
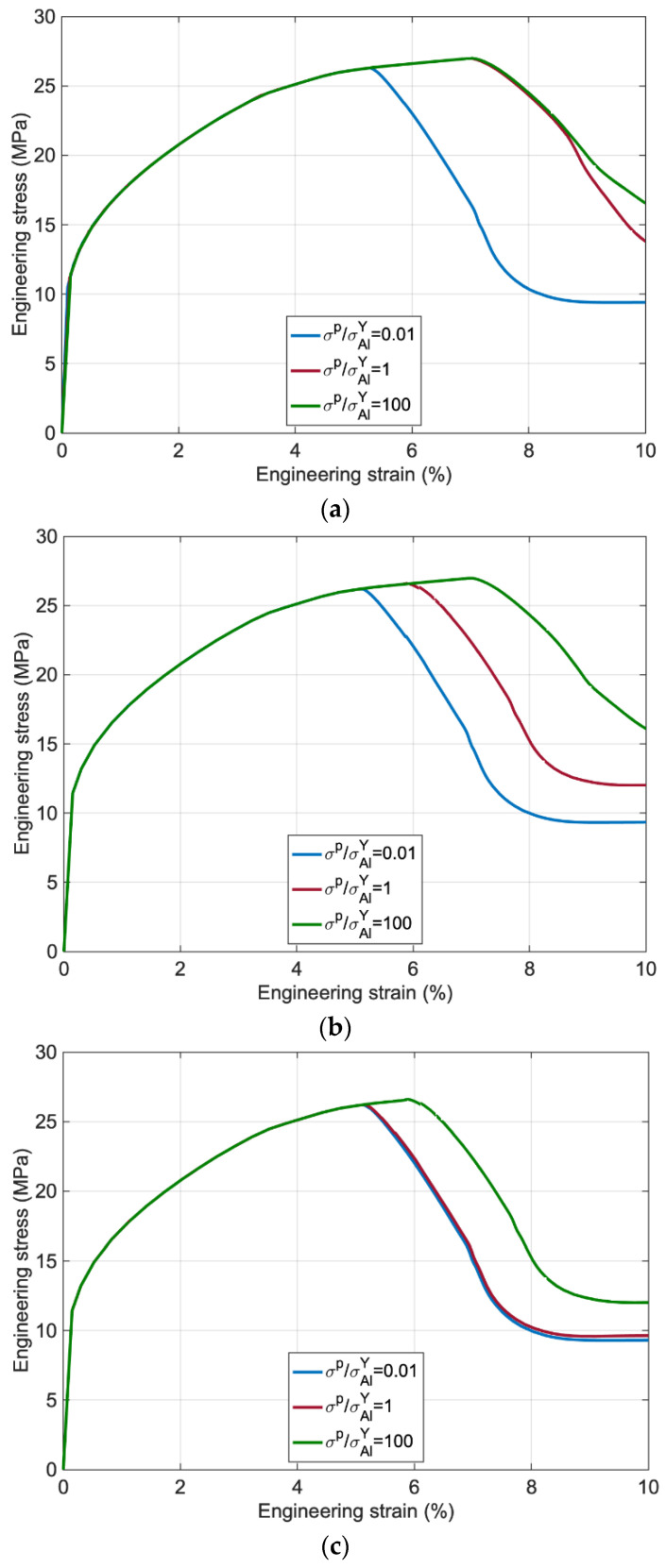
Engineering stress versus engineering strain for (**a**) $\delta^{p}/h=0.01$, (**b**) $\delta^{p}/h=1$ and (**c**) $\delta^{p}/h=100$.

The overall behavior of the material with different traction and separation values is provided in Figure 9. The results emphasize that a change in traction leads to different behavior, particularly in the softening branch, such that the higher traction retards the Al degradation, and by reducing the traction, the maximum stress reduces as well.

By increasing the separation quantities ($\delta^{p}/h$) from 0.01 to 100, the results highlight that if the separation is low, adopting high quantities for traction leads to the same behavior, while for higher separation, the laminate behavior is almost unaffected by lower tractions. 

The primary function of LDPE connected to a thin Al foil is to improve the Al foil’s final elongation. In order to attain this goal in a real-world application, the interfacial layer properties should be adjusted to have weak separation and medium traction properties.

## 4. Closing Remarks

This research concerns material systems made of thin metal foils and polymer ply coupling. In the present work, computational approaches were employed to investigate the mechanical behavior of thin laminated structures, considering a kind of imperfection and the interfacial layer effect. First, the interfacial layer properties were calibrated based on available uniaxial experimental tests on thin laminates, with and without crack. Calibrated parameters could reflect the nominal stress-strain behavior of plain and notched laminates closely comparable to experimental behavior. They could capture both maximum stress and the softening behavior after the degradation of the thin Al foil. 

Then, a wavy surface with a sinusoidal shape was defined on both the Al foil and polymer surfaces to investigate its influence on the overall behavior of the thin laminate and its failure mode. A numerical model consisting of Al foil coupled to the polymer under plane strain conditions was developed, and an interface layer between Al and polymer with zero-thickness cohesive elements characterized by T–S law (using calibrated parameters) was defined. The results based on the developed numerical models can be highlighted as:

- Among three cases (roughness on Al, LDPE and both), the wavy surface on only the LDPE layer led to the most critical condition such that the maximum level of stress decreased significantly when increasing the amplitude of the wavy interface.
- The failure mode of the laminate changes by varying the imperfection location and different failure modes observed by defining surface roughness on each and both layers.
- As the existence of roughness on the surface of each layer or both in the laminate is inevitable during manufacturing, the results of this study indicate the significant influence of this type of imperfection on the functionality of thin metal/polymer laminates, which is worthwhile in real-world applications.

As the interface layer properties play an important role in the laminate’s overall behavior, a parametric study was also performed, changing the traction and separation quantities in the T–S law. The results indicated that:

- At a constant separation, increasing tractions leads to higher stress and retarding the strain localization. On the other hand, at low separation, adopting higher traction results in almost the same mechanical behavior, and at high separation, the behavior of the laminate is unchanged by reducing the traction.
- Regarding the mentioned applications, these results can emphasize that adopting very high traction or very low separation does not necessarily lead to a strong interface behavior, but an optimum combination should be adopted.

## Figures and Tables

**Table 1:** Mechanical properties of Al foil and LDPE.

Type	Young’s Modulus (MPa)	Poisson Ratio (−)	Yield Limit (MPa)	*n* (−)
Al foil	46,000	0.3	35	0.015
LDPE	200	0.3	6.5	0.55

**Table 2:** Mechanical properties of the laminate.

Young’s Modulus (MPa)	Poisson Ratio (−)	Yield Limit (MPa)	*n* (−)
17,900	0.3	8	0.035

**Table 3:** Geometry details of the laminate.

Length (mm)	Width (mm)	Thickness (mm)
230	95	0.036

**Table 4:** Calibrated interfacial layer properties.

Initial Stiffness (MPa/mm)	Maximum Traction (MPa)	Ultimate Separation Displacement (mm)
17.9×10^6^	17.5	36

**Table 5:** Sequence of change in maximum traction and the final separation.

Maximum Traction Ratio $\left(\sigma^{p}/\sigma_{Al}^{Y}\right)$ (−)	Separation Ratio $\left(\delta^{p}/h\right)$ (−)
0.01	0.01
1	1
100	100

## Data Availability

Not applicable.

## References

[1] Shavit D. (2007). The developments of LEDs and SMD Electronics on transparent conductive Polyester film. Vac. Int..

[2] Suo Z., Vlassak J., Wagner S. (2005). Micromechanics of macroelectronics. China Particuology.

[3] Li T., Zhang Z., Michaux B. (2011). Competing failure mechanisms of thin metal films on polymer substrates under tension. Theor. Appl. Mech. Lett..

[4] Li T., Suo Z. (2007). Ductility of thin metal films on polymer substrates modulated by interfacial adhesion. Int. J. Solids Struct..

[5] Lu N., Suo Z., Vlassak J.J. (2010). The effect of film thickness on the failure strain of polymer-supported metal films. Acta Mater..

[6] Li T., Huang Z.Y., Xi Z.C., Lacour S.P., Wagner S., Suo Z. (2005). Delocalizing strain in a thin metal film on a polymer substrate. Mech. Mater..

[7] Xiang Y., Li T., Suo Z., Vlassak J.J. (2005). High ductility of a metal film adherent on a polymer substrate. Appl. Phys. Lett..

[8] Kamaliya P.K., Upadhyay S.H., Mallikarachchi H.M.Y.C. (2021). Investigation of wrinkling behaviour in the creased thin-film laminates. Int. J. Mech. Mater. Des..

[9] Shahmardani M., Ståhle P., Islam M.d.S., Kao-Walter S. (2020). Numerical Simulation of Buckling and Post-Buckling Behavior of a Central Notched Thin Aluminum Foil with Nonlinearity in Consideration. Metals.

[10] Islam M.S., Andreasson E., Kao-Walter S. (2019). Trouser tear testing of thin anisotropic polymer films and laminates. Int. J. Fract..

[11] Ståhle P., Shahmardani M., Kao-Walter S. (2020). On buckling and fracture of thin elastic-plastic foils. Procedia Struct. Integ..

[12] Nowak-Grzebyta J., Meijer F., Bula K., Stachowska E. (2020). Non-destructive Testing of Metal-Polymer Laminates with a Digital Holographic Vibrometer. J. Nondestruct. Eval..

[13] Kao-Walter S. (2004). On the Fracture of Thin Laminates. Ph.D. Thesis.

[14] Bolzon G., Shahmardani M., Liu R., Zappa E. (2017). Failure analysis of thin metal foils. Frat. Integrita Strutt..

[15] Chen S.H., Wang T.C., Kao-Walter S. (2003). A crack perpendicular to the bimaterial interface in finite solid. Int. J. Solids Struct..

[16] Andreasson E., Kao-Walter S., Ståhle P. (2014). Micro-mechanisms of a laminated packaging material during fracture. Eng. Fract. Mech..

[17] Kao-Walter S., Dahlström J., Karlsson T., Magnusson A. (2004). A study of the relation between the mechanical properties and the adhesion level in a laminated packaging material. Mech. Compos. Mater..

[18] Sharif U., Sun B., Islam M.S., Majeed K., Ibrahim D.S., Adewale O.O., Akhtar N., Zaki Z.I., El-Bahy Z.M. (2021). Fracture Toughness Analysis of Aluminum (Al) Foil and Its Adhesion with Low-Density Polyethylene (LPDE) in the Packing Industry. Coatings.

[19] Andreasson E., Abdulfeta J., Katangoori R.R. (2012). Is it possible to open beverage packages virtually? Physical tests in combination with virtual tests in Abaqus. SIMULIA Community Conference.

[20] Bolzon G., Shahmardani M. (2017). Macroscopic response and decohesion models of metal-polymer laminates. Eng. Trans..

[21] De SouzaNeto E.A., Peric D., Owen D.R.J. (2008). Computational Methods for Plasticity.

[22] Li B., Li Y., Su J. (2014). A combined interface element to simulate interfacial fracture of laminated shell structures. Compos. B Eng..

[23] Xie D., Waas A.M. (2006). Discrete cohesive zone model for mixed-mode fracture using finite element analysis. Eng. Fract. Mech..

[24] Xu W., Yang J.S., Lu T.J. (2011). Ductility of thin copper films on rough polymer substrates. Mater. Des..

